# The Interplay of Family Functioning and Impulsivity in Offending Patterns Among Incarcerated Adolescents

**DOI:** 10.3390/bs16060937

**Published:** 2026-06-06

**Authors:** Esma Altinel Acoglu, Ayşegül Efe, Sıddıka Songul Yalçın

**Affiliations:** 1 Department of Pediatrics, Ankara Etlik City Hospital, Ankara 06170, Türkiye; 2 Department of Child Psychiatry, Ankara Etlik City Hospital, Ankara 06170, Türkiye; 3 Department of Pediatrics, Faculty of Medicine, Hacettepe University, Ankara 06100, Türkiye

**Keywords:** juvenile delinquency, family functioning, impulsivity, offense patterns

## Abstract

This study examined whether family functioning and impulsivity differentiate offense categories or represent shared vulnerability factors among justice-involved adolescents. A cross-sectional survey was conducted in two juvenile correctional facilities and included 159 incarcerated male adolescents aged 13–18 years (mean 16.3 ± 1.1). Participants completed a case report form, the Family Assessment Scale (FAS), and the UPPS Impulsive Behavior Scale. Offenses were classified as property crimes (42%), crimes against persons (31%), sexual offenses (17%), and drug-related crimes (10%). Substance use was highly prevalent (smoking 86.8%; alcohol 61.0%), and 34.6% had experienced repeat incarceration, most frequently in property and drug-related offenses (*p* < 0.001). Peer influence was the most commonly reported reason for delinquency (44.7%). Family dysfunction was common across the sample, particularly in domains related to parental involvement and behavioral control with some variation across offense categories. In contrast, impulsivity levels were elevated but did not significantly differ between crime categories. These findings support a shared vulnerability perspective, suggesting that dysfunctional family environments and substance-related risk contexts operate across offenses, while impulsivity may represent a general risk rather than an offense-specific determinant. These results highlight the importance of family-centered and developmentally informed interventions in juvenile justice settings.

## 1. Introduction

Adolescence is a critical developmental period marked by rapid biological, psychological, and social transitions, during which vulnerability to risk-taking and antisocial behaviors, including juvenile delinquency, increases. While such behaviors may be temporary or situational for many adolescents, they can nonetheless lead to serious and long-lasting consequences, particularly when they result in involvement with the criminal justice system. Understanding the individual, familial, and environmental factors that contribute to juvenile delinquency and justice involvement is therefore essential for developing effective prevention and intervention strategies (Loeber & Farrington, 2012; Steinberg, 2017).

Adolescents who engage in juvenile delinquency require a developmentally informed approach that differs from that applied to adults (Sarı et al., 2019). During this stage, environmental influences, family dynamics, and behavioral characteristics such as impulsivity play an important role in shaping behavior (Ayar et al., 2022; Çiçek & Yalçin, 2023). Accordingly, examining delinquency within its broader developmental and social context is essential (Sarı et al., 2019).

Family structure and dynamics are consistently identified as key correlates of child behavior (Çiçek & Yalçin, 2023). Parenting practices such as monitoring, emotional responsiveness, role clarity and behavioral control are central to the development of self-regulation and social adaptation. Dysfunction in these domains has been associated with a range of adverse outcomes, including delinquent behavior (Peter & Nwadukwe, 2022). Family dynamics influence adolescent behavior through multiple interrelated mechanisms, including parental monitoring, emotional bonding, communication patterns, and behavioral regulation. Ineffective parental supervision and inconsistent discipline may limit adolescents’ ability to internalize social norms, increasing their susceptibility to delinquent behavior (Hoeve et al., 2009; Saladino et al., 2021). Similarly, poor emotional responsiveness and weak parent–child attachment can contribute to difficulties in emotion regulation, impulsivity, and affiliation with deviant peer groups (Gubbels et al., 2019; Saladino et al., 2021). Conversely, supportive and well-functioning family environments have been shown to act as protective factors by promoting self-control, social competence, and adaptive coping strategies (Hoeve et al., 2009). Non-intact families (e.g., parental separation, divorce, or parental bereavement), maternal employment, extended family living with many siblings, dysfunctional family relationships, inadequate parental supervision and discipline, discontinuation of education, psychiatric issues in the adolescent’s history, substance and alcohol use, and being male have been reported as significant risk factors for juvenile delinquency (Karataş, 2020; Patiz & Bayraktar, 2023; Vanassche et al., 2014). These factors often culminate in an adolescent’s susceptibility to delinquency, underscoring the importance of family functionality in mitigating these risks. The concept of family functionality, as introduced by Epstein et al. (1983), includes various dimensions such as problem-solving, communication, role distribution, emotional responsiveness, behavioral control, and general family functions. Families that effectively resolve problems together, maintain emotional bonds, exhibit care without overstepping boundaries, fulfill expected roles, and foster open and direct communication are considered healthy or functional (Ayar et al., 2024; Bulut, 1990; Epstein et al., 1983). In contrast, unhealthy family dynamics can have long-lasting detrimental effects on an adolescent’s development, potentially leading to delinquent behavior (Peter & Nwadukwe, 2022). These findings are consistent with international studies indicating that family disruption, poor parental supervision, and early school dropout are key predictors of delinquency across diverse cultural settings (Gubbels et al., 2019; Hoeve et al., 2009; Mwangangi, 2019).

Impulsive behavior has been strongly associated with juvenile offending, with adolescent offenders displaying higher levels of impulsivity compared to their non-offending peers (Carroll et al., 2006). Recent longitudinal findings further show that lower impulse control and higher sensation seeking are associated with increased offending during adolescence, underscoring the role of self-regulation processes in delinquent behavior (Wasserman et al., 2024). To better understand how impulsivity may relate to different patterns of delinquency, Whiteside and Lynam’s UPPS model (Urgency, lack of Premeditation, lack of Perseverance, and Sensation Seeking) conceptualizes impulsivity as a multidimensional construct into four facets: urgency (acting impulsively to relieve distress), lack of premeditation (acting without considering long-term consequences), lack of perseverance (difficulty maintaining focus on challenging tasks), and sensation seeking (seeking novel and risky experiences) (Whiteside & Lynam, 2001). This multidimensional perspective allows for a more nuanced understanding of the role of impulsivity in adolescent delinquent behaviors. Risky behaviors such as substance use, theft, bullying, physical violence, property damage, and attempted suicide are notably prevalent during the adolescent period (Akanni et al., 2017). However, evidence remains inconsistent regarding whether impulsivity differentiates specific offense types. Some studies have reported selective associations between impulsivity facets and specific crime types; for example, urgency has been linked to property crime and fraud, and lack of premeditation to property crime (Shin et al., 2016). However, this evidence is based on young adult samples aged 18–25, and comparable findings in adolescents remain limited. Evidence from adolescent offender samples also demonstrates a significant positive association between impulsivity and aggression, although these studies do not differentiate specific offense types (Srinivasan et al., 2022). These findings suggest that impulsivity may function as a general vulnerability factor rather than a consistent determinant of specific crime types.

To better understand whether risk factors are related to specific types of offenses or reflect broader patterns of delinquency, these relationships should be interpreted within established criminological frameworks (Gottfredson & Hirschi, 2022). These perspectives distinguish between offense specialization and offending versatility. These approaches suggest that differences in offense types may not arise from distinct causes, but rather reflect shared underlying vulnerabilities such as family dysfunction, poor supervision, and impulsivity. From this perspective, different offense categories can be seen as behavioral expressions of common risk environments. Evidence indicates that strict offense specialization among juvenile offenders is limited, with many adolescents showing involvement in multiple types of offending driven by general risk factors and cumulative environmental adversity (Eker & Mus, 2016). In line with general theories of crime, low self-control and weak social bonds increase the likelihood of engaging in various forms of offending rather than a single offense type (Gottfredson & Hirschi, 2022; Loeber & Farrington, 2012). Accordingly, specialization and versatility may vary across individuals and developmental stages, suggesting that offense categories alone may not fully explain the underlying mechanisms of juvenile delinquency. Within the Turkish socio-cultural context, examining dimensions of family functioning and behavioral vulnerability associated with juvenile offending may help clarify whether shared developmental risk factors underlie different offense types.

Most existing studies on adolescent delinquency focus on Western contexts, and little is known about how these risk factors manifest in settings such as Turkey (Abhishek & Balamurugan, 2024; Gatti et al., 2015; Loeber & Farrington, 2012). Within the socio-cultural framework of Turkey, extended kinship networks can provide emotional and financial support which buffer against hardship, yet may also facilitate the intergenerational transmission of antisocial norms when older relatives or peers are involved in criminal activities. In the Turkish socio-cultural context, traditional gender norms may encourage risk-taking and externalizing behaviors among boys, which may influence both the prevalence and diversity of delinquent acts (Çağlar, 2025; Deryol et al., 2021). The study focuses on male adolescents due to the structure of the participating juvenile facilities, which house only male youth.

The present study aimed to examine whether family functioning and impulsivity differ across offense types among justice-involved male adolescents, or whether these factors reflect broadly shared patterns across different forms of delinquency.

Grounded in developmental criminology and theories emphasizing offending versatility and shared vulnerability processes (Gottfredson & Hirschi, 2022; Loeber & Farrington, 2012), the study explored whether different offense categories are characterized by distinct psychosocial profiles or whether common developmental and environmental risk factors are observed across offense types. Specifically, the study sought to:

(1) Describe the distribution of offense types and associated individual and family characteristics;
(2) Assess differences in family functioning and impulsivity across offense categories;
(3) Evaluate whether observed differences persist after adjustment for key sociodemographic and behavioral factors.

Based on the existing literature and theoretical frameworks emphasizing offending versatility and shared developmental vulnerabilities (Gottfredson & Hirschi, 2022; Loeber & Farrington, 2012), we formulated the following hypotheses:

(1) Adolescents involved in different offense categories would demonstrate variation in specific dimensions of family functioning, particularly in domains related to parental involvement, behavioral regulation, and family organization;
(2) Impulsivity would be elevated across the sample regardless of offense category, supporting its role as a generalized developmental vulnerability rather than an offense-specific characteristic;
(3) After adjustment for contextual factors such as school attendance, alcohol use, and family history of incarceration, specific family functioning subscales would show limited differentiation, whereas general family functioning would remain a significant and independent associate across offense categories.

By enhancing our understanding of these interrelated factors, the study helps to inform prevention and intervention strategies targeting vulnerable youth, reduce recidivism, and support healthier developmental outcomes through tailored, family-based approaches.

## 2. Materials and Methods

### 2.1. Study Design and Setting

This cross-sectional descriptive study was conducted as a voluntary survey between June and September 2024 at the Ankara Children’s Education Center and the Sincan Children and Youth Closed Penal Institution. Necessary permissions were obtained from the Ministry of Justice Prisons and Detention Houses General Directorate. The Ankara Children’s Education Center is a semi-secure facility focusing on rehabilitation and educational programs, whereas the Sincan Children and Youth Closed Penal Institution is a secure correctional facility designed for juveniles convicted of more severe offenses. Adolescents are placed in these institutions by court order, and both facilities operate under the Ministry of Justice, with multidisciplinary teams providing psychological, social, and educational support. Ethical approval was granted by the Ankara Etlik City Hospital Ethics Committee (Approval Number: AEŞH-BADEK-2024-015). Written informed consent was obtained from all participants before their inclusion in the study. Participation was entirely voluntary, and it was explicitly stated that choosing not to participate would have no influence on their current legal status or institutional treatment. The authors did not access any information that could identify individual participants during or after data collection. Special attention was given to the protection of this vulnerable population, ensuring voluntary participation, confidentiality, and the absence of any perceived coercion throughout the study.

### 2.2. Participants and Data Collection

The Ankara Children’s Education Center and Sincan Juvenile and Youth Closed Prison differ in their primary function: the Children’s Education Center mainly accommodates sentenced adolescents, whereas the Children and Youth Closed Penal Institution primarily houses detained youth, along with a smaller number of sentenced individuals. Therefore, the populations are partially overlapping rather than strictly distinct. There was a total of 346 individuals in the two institutions. Under certain conditions, such as continuation of education or institutional employment, individuals may remain in these facilities up to the age of 21. In the present study, individuals older than 18 years (*n* = 130) were excluded to ensure a developmentally homogeneous adolescent sample. Of the remaining eligible adolescents (*n* = 216), 166 provided written informed consent. After excluding seven participants due to incomplete forms, the final sample consisted of 159 adolescents.

The participating institutions were juvenile justice facilities housing male adolescents. Although female-only institutions exist in the same city, they were not included, as the number of female adolescents was insufficient for statistically meaningful gender-based comparisons.

Sociodemographic data and offense-related information were collected via structured Case Report Form (CRF) through face-to-face interviews conducted by trained institution staff (pedagogue and social service specialist). The Family Assessment Scale (FAS) and UPPS Impulsive Behavior Scale were self-administered by participants under supervision, ensuring independent responses. Clarification was provided when necessary without influencing responses. All interviews and questionnaire administrations were conducted individually in a private setting. No group administration was used. The completion process took approximately 20–30 min per participant.

Prior to data collection, participants were provided with detailed information about the study, including its voluntary nature and confidentiality procedures, and written informed consent was obtained. To ensure confidentiality, participants placed their completed forms in sealed envelopes and submitted them into a secure collection box accessible only to the research team.

### 2.3. Data Collection Instruments

Three instruments were used in this study.

#### 2.3.1. Case Report Form (CRF)

The CRF was developed based on an extensive literature review (Nisar et al., 2015; Peter & Nwadukwe, 2022; Sarı et al., 2019) to collect detailed information on sociodemographic characteristics and risk factors associated with delinquent behavior. It consists of 47 items covering domains such as family structure, education, psychiatric and physical health, substance use, and criminal history, and includes both structured and semi-open-ended questions. A pilot test with 10 adolescents was conducted to evaluate clarity and comprehensibility, after which necessary revisions were made to improve the instrument.

#### 2.3.2. Family Assessment Scale (FAS)

The FAS is based on the McMaster Family Functioning Model (Epstein et al., 1983) and was adapted to Turkish by Bulut (1990). It evaluates family functioning across seven subscales: problem-solving, communication, roles, emotional responsiveness, attention, behavior control, and general family functioning. For instance, the behavior control subscale evaluates the family’s approach to regulating and monitoring behaviors, while emotional responsiveness measures the ability of family members to respond to emotional needs. The scale comprises 60 items rated on a 4-point Likert scale ranging from 1 (healthy) to 4 (unhealthy). Subscale scores are calculated as the mean of the responses, with a threshold of 2 distinguishing between healthy (<2) and unhealthy (≥2) family functioning. Higher scores indicate impaired family functioning. Cronbach’s alpha values for the subscales, as reported by Bulut, ranged from 0.38 to 0.86. In the current study, internal consistency varied across subscales. The attention subscale showed very low internal consistency (α < 0.20), and the behavior control subscale also demonstrated low reliability (α = 0.25). These findings indicate that these subscales may not reliably measure a coherent construct in this sample. Therefore, results related to these subscales should be interpreted with caution. Rather than representing specific constructs such as parental neglect or behavioral control deficits, these domains are better understood as reflecting general patterns of perceived family functioning with limited psychometric reliability in this context. The remaining subscales demonstrated moderate internal consistency (α > 0.50), suggesting acceptable reliability for exploratory analysis (Table 1). It should also be noted that Cronbach’s alpha reflects internal consistency rather than underlying factor structure; thus, further psychometric evaluation, including factor analytic approaches, would be needed to confirm the dimensional structure of the scale in this population.

#### 2.3.3. UPPS Impulsive Behavior Scale

The UPPS Impulsive Behavior Scale is a 45-item measure developed by Whiteside and Lynam (2001) to assess impulsivity using a four-factor model. The Turkish adaptation by Yargıç et al. (2011) demonstrated good reliability and validity. The scale is scored on a 4-point Likert scale ranging from 1 (Strongly Disagree) to 4 (Strongly Agree), with higher scores indicating greater impulsivity. The four subscales of the UPPS include Lack of Premeditation, Urgency, Sensation Seeking, and Lack of Perseverance. Cronbach’s alpha for the total UPPS score in this study was 0.86, and all subscales had internal consistency values exceeding 0.7, indicating good reliability (Table 1).

### 2.4. Classification of Criminal Offenses

The offenses committed by the participants were categorized into four groups. Offense classification was based on the most serious current offense leading to institutional placement. In cases where multiple charges were present, only the most serious offense was recorded for analysis (Bisogno et al., 2015; Durrant, 2021):

- Property Crimes: Theft, burglary, robbery, vandalism, arson;
- Crimes Against Persons: Assault, homicide, battery;
- Sexual Offenses: Sexual assault, rape, child molestation;
- Drug-Related Crimes: Drug possession, trafficking, distribution.

### 2.5. Statistical Analysis

Data were analyzed using IBM SPSS Statistics for Windows, Version 23.0. Armonk, NY, USA: IBM Corp. The normality of continuous variables was assessed using kurtosis, skewness, histograms, and the Kolmogorov–Smirnov test. Descriptive statistics were reported as frequencies (*n*, %), means ± standard deviation (SD), and medians with interquartile ranges (IQR) as appropriate based on data distribution.

Since the UPPS subscales did not follow a normal distribution, impulsivity scores were categorized into tertile groups (low, moderate, and high) using the 33rd and 66th percentiles. However, given the potential loss of information associated with categorization, primary interpretations were based on continuous variables and summarized using medians and interquartile ranges (IQR).

Differences between categorical variables were assessed using the Chi-square test, or Fisher’s exact test when expected cell counts were low. When overall group differences were statistically significant, post-hoc pairwise comparisons were conducted using Bonferroni correction to account for multiple comparisons.

Consolidated reasons were generated by grouping conceptually similar self-reported responses to enhance interpretability and facilitate statistical comparison across offense categories.

One-way ANOVA was used to compare mean differences across multiple groups. Kruskal–Wallis ANOVA was applied for non-normally distributed variables.

Descriptive analyses were conducted to characterize offense-specific psychosocial patterns, whereas multivariable models were used to evaluate whether these differences persisted after adjustment for shared developmental and contextual vulnerabilities.

To examine whether differences in family functioning across offense types remained after adjustment for potential confounders, generalized linear models (GLMs) with normal distribution and identity link function were applied. Offense type was included as a fixed factor, and selected covariates (age, family history of incarceration, adolescents’ alcohol use, and school attendance) were entered into the models. Adjusted group differences were presented as estimated marginal means with 95% Wald confidence intervals. Post-hoc pairwise comparisons were conducted using Bonferroni correction.

Internal consistency of the scales was evaluated using Cronbach’s alpha. Given that Cronbach’s alpha reflects internal consistency rather than factor structure, results were interpreted accordingly.

A *p*-value of <0.05 was considered statistically significant.

## 3. Results

### 3.1. Individual Characteristics

The mean age of the participants was 16.3 ± 1.1 years (range: 13–18 years), with 15.7% being 15 years old or younger. Before incarceration, the school attendance rate was 54.7%, and the employment rate was 81.8%, referring to adolescents’ engagement in paid work prior to incarceration rather than parental employment. School attendance differed significantly across offense types (*p* = 0.018), being highest among adolescents convicted of sexual crimes (77.8%) and lowest among those involved in property crimes (42.4%). Employment rates did not differ significantly by offense type (*p* = 0.060), although the highest rate was observed in property crimes (90.9%) and the lowest in crimes against persons (72.0%) (Table 2).

The prevalence of smoking was 86.8%, and 22.6% of participants reported initiating smoking between ages 5 and 9, indicating early substance exposure. Alcohol consumption was reported by 61% of participants, with the highest prevalence among those committing drug-related crimes.

### 3.2. Offense Types

Crimes were classified into four categories: property crimes (42%), crimes against persons (31%), sexual crimes (17%), and drug-related crimes (10%) (Table 2). Among those committing crimes against persons, 44% (*n* = 22) were involved in murder, including one case combined with a sexual offense. Other offenses in this category included wounding, attempted murder, and deprivation of liberty. Notably, 20% of those convicted of crimes against persons were 15 years old or younger.

### 3.3. Family and Living Conditions

Among the participants, 7.6% had at least one deceased parent, while 18.2% reported having a stepparent. Overall, 59.7% lived with both parents, whereas 3.1% (*n* = 6) lived outside the home, including five on the streets and one in a cemetery.

The number of siblings showed a significant association with crime type (*p* = 0.001). Adolescents involved in property crimes (89.4%) and drug-related crimes (100%) were more likely to have three or more siblings compared to those convicted of sexual offenses.

A family history of incarceration was reported by 40.3% of participants, with the highest rates observed in the drug-related crime group and the lowest among those convicted of sexual offenses (*p* = 0.001).

More than half of the participants’ mothers smoked, with significant differences by crime type: maternal smoking was most prevalent among adolescents involved in drug-related crimes and least common in sexual offenses (*p* = 0.031).

### 3.4. Recidivism and Reasons for Delinquency

Recidivism was reported in 34.6% of participants and differed significantly by offense type (*p* < 0.001), being more frequent among adolescents involved in property and drug-related crimes compared to other groups (Table 3). Peer influence was the most frequently cited reason for delinquency (44.7%), followed by family and economic difficulties (10.1%) and psychological issues (6.9%). For property and crimes against persons, peer influence and social environment were the dominant factors.

Drug-related crimes showed distinct patterns, being significantly associated with childhood alcohol use (*p*< 0.001) and maternal smoking (*p* = 0.031). Among these offenders, drug use and peer influence were the most frequently reported reasons, while none mentioned psychological issues. In contrast, 51.9% of adolescents convicted of sexual offenses either did not specify a reason or stated, “I don’t know’’ (Table 3).

### 3.5. Family Functioning

Significant associations were found between offense type and the FAS subscales of Problem-Solving, Roles, Emotional Responses, and General functioning (Table 4). Adolescents convicted of sexual offenses showed the highest functional scores in the Roles (66.7%) and General functioning (70.4%) subscales, whereas those involved in property and drug-related crimes had markedly lower scores (*p* = 0.001 and *p* = 0.047, respectively).

Overall, 92.5% of participants had pathological scores on the “Showing Necessary Attention” subscale, and more than two-thirds scored in the dysfunctional range for Communication and Behavioral Control. These findings indicate substantial deficits in parental attention, guidance, and emotional support across the sample.

The behavior control subscale evaluates how families regulate and monitor behaviors, while emotional responsiveness measures the ability of family members to respond to emotional needs. Adolescents involved in property and drug-related crimes appeared to come from less organized households with weaker role distribution and problem-solving abilities, whereas those with sexual offenses demonstrated relatively better family cohesion despite their offenses.

### 3.6. Impulsivity and Crime Type

No significant differences were observed in UPPS impulsivity subscale scores across crime types, whether analyzing median values or tertile distributions (Table 4). Although impulsivity scores did not differ significantly by crime type, the median scores for urgency and sensation seeking indicate generally elevated impulsivity levels across the sample, suggesting a high baseline risk.

### 3.7. Generalized Linear Model Analysis of Family Functioning Across Offense Types

Adjusted analyses using generalized linear models showed that most FAS subscales did not differ significantly across offense types (Table 5). Specifically, no statistically significant differences were observed for problem-solving (*p* = 0.392), communication (*p* = 0.834), roles (*p* = 0.334), emotional responsiveness (*p* = 0.223), or behavioral control (*p* = 0.183) after adjustment for age, family history of incarceration, adolescents’ alcohol use, and school attendance. Although the overall model indicated a borderline difference for the “showing necessary attention” subscale (*p* = 0.049), post-hoc pairwise comparisons were not statistically significant after correction. In contrast, general family functioning remained significantly different across offense types (*p* = 0.021), with adolescents involved in sexual offenses demonstrating better overall functioning.

## 4. Discussion

This cross-sectional study examined whether family functioning and impulsivity differ across offense types among justice-involved male adolescents or reflect shared patterns across different forms of delinquency. The findings indicate that family dysfunction, substance-related risk factors, and school disengagement were prevalent across offense categories, suggesting broadly shared risk contexts rather than clearly distinct, offense-specific profiles. Overall, the findings suggest that different offense types may share common developmental and environmental risk factors rather than reflecting clearly distinct psychosocial profiles. In Turkish society, boys are typically granted greater freedom of movement and experience lower levels of parental supervision than girls. This may increase their exposure to deviant peers and environmental risks (Karataş, 2020). From this perspective, socio-cultural factors may play an important role in the assessment of risk factors associated with juvenile delinquency.

Although several FAS subscales differed across offense types in unadjusted analyses, these differences were largely attenuated after adjustment. Generalized linear models revealed that only general family functioning remained significantly different across offense categories after controlling for age, school attendance, adolescents’ alcohol use, and family history of incarceration. Specifically, adolescents involved in sexual offenses demonstrated better adjusted general family functioning scores compared to other groups. This may suggest that overall family functioning may represent a more robust dimension associated with offense patterns than individual subdomains, although these findings should be interpreted cautiously given the study design.

Across the sample, family dysfunction was highly prevalent, with substantial impairments observed in multiple domains. Approximately 40% of the adolescents in this study came from family structures involving divorced parents, at least one deceased parent, or the presence of a stepparent. Notably, a large proportion of participants reported difficulties in perceived parental attention and involvement, indicating that limitations in parental engagement may represent a common contextual feature among justice-involved adolescents. These findings are consistent with prior research highlighting the role of disrupted family processes, inadequate supervision, and poor communication as cross-cutting correlates of juvenile delinquency across diverse settings (Ataç, 2023; Hoeve et al., 2009; Peter & Nwadukwe, 2022; Reeta & Singh, 2020; Saladino et al., 2021). From a theoretical perspective, this pattern aligns with frameworks emphasizing offending versatility, which propose that general family and environmental vulnerabilities may underlie multiple forms of delinquent behavior rather than distinct, offense-specific pathways (Eker & Mus, 2016; Loeber & Farrington, 2012). A child’s family environment plays a pivotal role in shaping law-abiding behavior, providing a foundation for learning how to manage inappropriate actions, develop tolerance, and respect others’ rights (Nisar et al., 2015).

The school dropout rate in this study was 45.3%, with the highest rates observed among adolescents convicted of property crimes, followed by those involved in drug-related crimes. Employment before incarceration was also most common among property offenders. These findings suggest that socioeconomic disadvantages, limited access to quality education, and early entry into the workforce are key factors contributing to juvenile delinquency. Prior studies have similarly reported that school disengagement, combined with financial hardships, parental neglect, or exposure to domestic violence, increases the risk of delinquency by pushing adolescence toward early employment and peer groups that normalize delinquency (Abhishek & Balamurugan, 2024; Atar et al., 2016; Gubbels et al., 2019). Ensuring continued school attendance and providing educational support to at-risk youth could be crucial for prevention.

Drug crimes were strongly associated with parental smoking, having more siblings, recidivism, and family members with incarceration histories. Adolescents involved in drug crimes exhibited the highest rates of early alcohol and tobacco use, with nearly half starting to smoke before age 10. Adolescence is a period of heightened vulnerability to risky behaviors, particularly among those from dysfunctional families, with peer influence playing a significant role (Kam & Middleton, 2013; Myers & Kelly, 2006; Vanassche et al., 2014). Early substance use is linked to poor school performance, delinquency, and progression to other drugs (Hoffman et al., 2001; Myers & Brown, 1994), while alcohol and drug use are known triggers for aggression and criminal activities, including theft and violence (Gatti et al., 2015; Massarwi & Khoury-Kassabri, 2017; Saladino et al., 2021). Parental incarceration further increases externalizing behaviors and substance abuse risks (Furr-Holden et al., 2011; Ruhland et al., 2020; Saladino et al., 2021). These findings demonstrate the intertwined nature of substance use, family dysfunction, and recidivism in this group.

Adolescents involved in sexual crimes showed relatively better family functioning scores compared with other offense groups, particularly in general functioning. However, this should not be interpreted as indicating an absence of family-related vulnerability. Rather, it may suggest that sexual offending among adolescents is influenced by more complex individual, relational, developmental, and contextual mechanisms that are not fully captured by global family functioning measures. Sexual crimes pose serious societal challenges, with long-term consequences for both victims and communities (Starzyk & Marshall, 2003). Previous research highlights links between insecure attachment, childhood maltreatment, and the risk of sexual offending (Grady et al., 2017; Miner et al., 2016; Naramore et al., 2017). A lack of emotional support and parental engagement may foster feelings of loneliness and social exclusion, increasing the likelihood of antisocial behaviors. In our study, more than half of the participants who committed sexual offenses did not specify a reason or stated that they did not know, likely reflecting the stigma and shame associated with these crimes. Peer influence remained the most commonly reported factor (44.4%), suggesting that inadequate familial support may heighten vulnerability to negative peer dynamics.

Property crimes were the most common offense type in our study (42%), followed by crimes against persons (31%). This finding is consistent with international reports indicating that property crimes are frequently the leading offenses among adolescents, largely influenced by socioeconomic challenges and peer dynamics (Abhishek & Balamurugan, 2024; Hoeve et al., 2009; Sullivan et al., 2019).

Property offenders in our study exhibited the highest rates of school dropout (45.8%), employment before incarceration (90.9%), and living outside the home (6.1%). School disengagement and limited educational opportunities are known to push adolescents toward antisocial peer groups and increase the likelihood of delinquency, particularly in socially disadvantaged communities (Naramore et al., 2017; Sullivan et al., 2019). Economic hardship, unstable family environments, and early entry into precarious work further compound this risk (Abhishek & Balamurugan, 2024; Naramore et al., 2017; Payne et al., 2003). Peer influence was the most commonly cited reason for property crimes (54.5%), followed by family and economic difficulties (15.2%). Family functioning was poor across all FAS subscales, with the lowest scores observed in the “Showing Necessary Attention” subscale (7.6%). Lack of parental supervision, ineffective communication, and family conflict have been identified as key predictors of property-related delinquency (Hoeve et al., 2009; McGloin & Thomas, 2019; Sullivan et al., 2019). Therefore, prevention efforts should focus on strengthening family bonds, promoting positive peer interactions, and supporting educational engagement.

Among those convicted of crimes against persons, family functioning scores were also lowest, with “Showing Necessary Attention” (6%), communication (32%), behavioral control (34%), and problem-solving (38%) being particularly impaired. Previous research has shown that children raised in single-parent or stepfamilies are more likely to engage in various delinquent activities, such as truancy, substance use, and running away from home (Brown & Rinelli, 2010; Hogan & Kitagawa, 1985; Song et al., 2012; Schroeder et al., 2010). In the present study, 44% of crimes against persons were murder cases. Peer influence (40%) and psychological problems (16%) were the most frequently reported reasons for crimes against persons. Heide’s longitudinal study on juvenile homicide offenders highlighted that such acts often arise from a combination of psychological stressors, including unresolved trauma and identity struggles, and sociological factors, such as association with deviant peers and exposure to violence in disadvantaged neighborhoods (Heide, 2021). A systematic review of juvenile homicide studies identified key risk factors, including poor executive functioning, mental health issues, prior contact with the justice system, and gang involvement, all of which interact with environmental influences to increase the risk of violent acts (Gerard et al., 2014). These findings emphasize the multifactorial nature of violent crimes and the importance of early interventions addressing both family and social risk factors.

Impulsivity is a characteristic frequently observed during adolescence and is associated with an increased likelihood of engaging in risky behaviors. Among juvenile offenders, impulsivity is known to heighten the probability of committing crimes due to a tendency for spontaneous and unplanned actions (Carroll et al., 2006). Studies demonstrating the relationship between substance abuse and impulsivity highlight the potential role of substance use in exacerbating impulsive tendencies, which in turn may contribute to delinquency (Saladino et al., 2021; Seker et al., 2021). In contrast, impulsivity scores did not significantly differ across offense categories. This pattern may indicate that impulsivity functions as a shared developmental vulnerability across delinquent behaviors rather than as an offense-specific mechanism. Within the framework of offending versatility, elevated impulsivity may increase general susceptibility to risk-taking and antisocial behavior across multiple offense types. This may help explain why impulsivity showed limited differentiation across offense categories despite its overall elevation within the sample.

## 5. Limitations and Strengths

Despite the rich data obtained from a hard-to-reach population, several limitations exist. The cross-sectional design precludes causal inferences. The relatively small size of some offense subgroups, particularly the drug-related offense group, limited statistical power and restricted the complexity of multivariable analyses. Although adjusted generalized linear models were conducted, these analyses were exploratory and should be interpreted cautiously.

The sample consists only of male participants from specific penal institutions in Ankara, limiting the generalizability of the findings to female juveniles or those from different cultural and geographical backgrounds within the broader context of Turkish youth. Additionally, recall bias and social desirability effects may have influenced survey responses. Although participation was voluntary and confidentiality procedures were emphasized, the inherent conditions of institutional oversight in detention settings may have subtly influenced participants’ perceptions of voluntariness. Consequently, the potential for social desirability bias—especially regarding sensitive reports of family dysfunction and substance use under institutional supervision—cannot be entirely dismissed. While the study sheds light on family dynamics, other crucial factors, such as childhood trauma, peer relationships, and attachment styles, were not examined.

Some subscales of the FAS demonstrated low internal consistency in this sample, limiting the interpretability of subscale-level findings. In the present study, our primary aim was not to validate the factor structure of the FAS but to use an established instrument in a specific clinical population. Due to the relatively limited sample size and the exploratory nature of the analyses, confirmatory factor analysis was not conducted. Crucially, these psychometric constraints further justify our analytical focus on the ‘General Functioning’ dimension within the generalized linear models, as it provided a more robust and stable composite metric for theory-driven modeling than the individual subscales. Therefore, findings related to specific family functioning domains should be interpreted with caution.

Despite these limitations, the study provides valuable insights into the role of family structure and impulsivity in juvenile delinquency. It highlights the significance of dysfunctional family dynamics and socioeconomic factors in shaping delinquent behavior. The findings contribute to the existing literature and offer a foundation for future research and intervention strategies.

Future longitudinal research is needed to determine whether improvements in general family functioning can actively reduce the versatility of criminal behavior over time.

## 6. Conclusions

Juvenile delinquency is a critical factor in ensuring that future generations grow up to be healthy, productive, and socially beneficial individuals. Preserving social stability and breaking the cycle of crime require a proper understanding of the underlying causes that lead children into delinquency, as well as timely and appropriate interventions. This study is based on rich and rarely accessible data derived from a vulnerable and scientifically understudied group. In this respect, it offers important contributions to both the academic literature and policy development within the juvenile justice system. Children who experience poverty, disrupted family structures, substance use, school dropout, and early employment are more vulnerable to risky behaviors. Dysfunctional family dynamics, such as parental indifference, lack of supervision, poor family communication, and inadequate problem-solving skills, can lead children toward risky behaviors and criminal tendencies. The lowest proportion of functional families was observed among drug offenders, suggesting that such families may be ineffective in preventing delinquency and supporting positive development. Socioeconomic difficulties and peer influence appear to be major contributing factors, particularly in property and drug-related crimes. The higher rate of functional families in sexual offenses may indicate that these crimes are often associated with more complex family or individual dynamics.

The high prevalence of smoking and alcohol use among incarcerated children indicates that substance use is a significant risk factor and a potential gateway to delinquency. Although impulsivity scores did not differ significantly across crime types, generally elevated levels suggest that juvenile delinquency should be examined not only through environmental lenses but also with attention to individual traits. Disrupted family structures, such as having a deceased parent or living with a stepparent, as well as extreme living conditions like homelessness, increase vulnerability to crime, especially violent crimes against individuals. The strong association between parental incarceration and children’s involvement in criminal activity reflects the intergenerational transmission of crime. It is essential to avoid blaming the child alone and instead to evaluate the impact of their surrounding environment on their behavior. To effectively prevent youth delinquency, public policies must address the complex familial, social, and socioeconomic factors that contribute to delinquency and aim to foster stable and supportive family environments. Providing at-risk children with access to quality education and essential resources can significantly reduce their likelihood of turning to crime as a means of survival.

Taken together, these findings suggest that family dysfunction, school disengagement, substance use, and family history of incarceration may represent shared developmental and environmental vulnerabilities underlying different offense patterns among justice-involved adolescents. Adjusted analyses suggest that general family functioning, rather than specific FAS subdomains, may be the most robust family-related dimension associated with offense categories. However, these findings should be interpreted cautiously due to the cross-sectional design, small subgroup sizes, and psychometric limitations of some FAS subscales.

## Figures and Tables

**Table 1:** Impulsive Behavior Scale and Family Assessment Scale subscale scores and Cronbach’s alpha values.

	Item No	Mean	Min	Perc			Max	Cronbach’s Alpha	
				25	50	75		Current Study	Yargıç et al. (2011)
Impulsive Behavior Scale									
Premeditation	11	21.4	11.0	17.0	22.0	26.0	44.0	0.84	0.86
Urgency	12	28.6	12.0	22.0	29.0	34.0	48.0	0.84	0.80
Sensation Seeking	12	27.5	12.0	23.0	28.0	31.0	48.0	0.74	0.85
Lack of Perseverance	10	20.8	10.0	16.0	22.0	25.0	37.0	0.74	0.80
UPPS Total Score	45							0.86	0.85
Family Assessment Scale *									Bulut (1990)
Problem-solving	6	2.08	1.00	1.67	2.00	2.50	4.00	0.63	0.80
Communication	9	2.14	1.11	1.78	2.11	2.56	3.56	0.55	0.71
Roles	11	2.11	1.27	1.82	2.09	2.45	3.45	0.52	0.42
Emotional responses	6	2.14	1.00	1.67	2.17	2.67	3.83	0.59	0.59
Showing necessary attention	7	2.55	1.71	2.29	2.57	2.71	3.57	−0.12	0.38
Behavior control	9	2.16	1.33	1.78	2.22	2.44	3.22	0.25	0.52
General functioning	12	1.97	1.00	1.50	2.00	2.42	3.42	0.78	0.86
FAS_overall	60							0.87	

* Subscale scores were divided into item number; therefore, the max score can be 4. UPPS: Urgency, lack of Premeditation, lack of Perseverance, Sensation Seeking.

**Table 2:** Associations of Individual and Family Characteristics With Crime Types Among Adolescents.

	Overall	Property Crimes	Crimes Against Persons	Sexual Offenses	Drug-Related Crimes	*p*
*n*	159	66	50	27	16	
Adolescent Characteristics						
Age ≤15 years	15.7	13.6	20.0	18.5	6.3	0.542 ^#^
Age (years)	16.3 ± 1.1	16.3 ± 0.9	16.2 ± 1.2	16.2 ± 1.2	16.2 ± 1.0	0.930 *
Previously attended school	54.7	42.4 ^a^	58.0 ^ab^	77.8 ^b^	56.2 ^ab^	0.018 ^#^
Previously employed	81.8	90.9	72.0	81.5	75.0	0.060 ^#^
Living Situation						
● Home	96.9	93.9	98.0	100.0	100.0	0.327 ^#^
● Outside the home	3.1	6.1	2.0	0.0	0.0	
Out-of-Family Care						
● None	79.2	75.8	76.0	92.6	81.2	0.287 ^#^
● At least 1 year	20.8	24.2	24.0	7.4	18.8	
Parental Status						
At least one deceased parent	7.6	7.6	12.0	3.7	6.2	0.614 ^#^
Presence of a stepparent	18.2	15.2	26.0	14.8	12.5	0.391 ^#^
Parents living together	59.7	62.1	52.0	66.7	62.5	0.574 ^#^
Number of Siblings						
● 1–2	20.8	10.6 ^a^	30.0 ^b^	40.7 ^b^	0.0 ^a^	0.001 ^#^
● 3–4	50.9	51.5	50.0	51.9	50.0	
● ≥5	28.3	37.9 ^a^	20.0 ^b^	7.4 ^b^	50.0 ^a^	
Maternal Education						
● No schooling/Unknown	31.4	37.9	28.0	22.2	31.2	0.175 ^#^
● Primary school	22.0	21.2	30.0	11.1	18.8	
● Middle school	26.4	24.2	18.0	37.0	43.8	
● High school/University	20.1	16.7	24.0	29.6	6.2	
Paternal Education						
● No schooling/Unknown	27.7	33.3	28.0	11.1	31.2	0.116 ^#^
● Primary school	23.9	19.7	30.0	14.8	37.5	
● Middle school	29.6	31.8	26.0	37.0	18.8	
● High school/University	18.9	15.2	16.0	37.0	12.5	
Parental Employment						
●Mother employed	31.4	26.6	36.2	33.3	33.3	0.737 ^#^
● Father employed	80.1	77.0	85.1	88.9	62.5	0.137 ^#^
Adolescent Smoking Behavior						
Smoker	86.8	90.9	84.0	77.8	93.8	0.272 ^#^
Age at first smoking experience						
● None	13.2	9.1	16.0	22.2	6.2	0.43 ^#^
● 5–9 years	22.6	27.3 ^a^	10.0 ^b^	22.2 ^ab^	43.8 ^a^	
● 10–12 years	30.8	28.8	36.0	18.5	43.8	
● ≥13 years	33.3	34.8 ^a^	38.0 ^a^	37.0 ^a^	6.2 ^b^	
Daily cigarette consumption						
● 0	13.8	9.1	18.0	22.2	6.3	0.121 ^#^
● 1–19	21.4	19.7	28.0	25.9	0.0	
● 20–39	39.6	47.0	30.0	33.3	50.0	
● ≥40	25.2	24.2	24.0	18.5	43.8	
Adolescents’ alcohol consumption	61.0	71.2 ^ab^	56.0 ^b^	25.9 ^c^	93.8 ^a^	<0.001 ^#^
Mother smokes cigarette	54.7	59.1 ^ab^	50.0 ^b^	37.0 ^b^	81.2 ^a^	0.031 ^#^
Father smokes cigarette	80.3	84.4	74.0	74.1	93.8	0.220 ^#^
Mother consumes alcohol	3.1	4.5	4.0	0.0	0.0	0.585 ^#^
Father consumes alcohol	22.9	28.1	24.0	7.4	25.0	0.193 ^#^

Values are given mean ± standard deviation or percentage. ^#^ Chi-square test; * one-way ANOVA. ^a,b,c^ Values having different letters in the same row were different with Bonferroni correction.

**Table 3:** Criminal Characteristics of Adolescents by Crime Type.

	Overall	Property Crimes	Crimes Against Persons	Sexual Offenses	Drug-Related Crimes	*p*
*n*	159	66	50	27	16	
Crime Characteristics of Adolescents						
Number of incarcerations						
1	65.4	50.0 ^a^	78.0 ^b^	96.3 ^c^	37.5 ^a^	<0.001 ^#^
2–3	15.7	15.2 ^a^	14.0 ^a^	3.7 ^a^	43.8 ^b^	
≥4	18.9	34.8 ^a^	8.0 ^bc^	0.0 ^c^	18.8 ^ab^	
Reason for Delinquency						
Peer influence	35.8	47.0 ^a^	28.0 ^b^	37.0 ^ab^	12.5 ^b^	<0.001 ^#^
Influence of social environment	8.8	7.6	12.0	7.4	6.2	
Family-related factors	4.4	6.1	4.0	0.0	6.2	
Economic difficulties	5.7	9.1	4.0	0.0	6.2	
Psychological reasons	6.9	3.0 ^a^	16.0 ^b^	3.7 ^ab^	0.0 ^a^	
Drug use	6.3	0.0 ^a^	2.0 ^a^	0.0 ^a^	56.2 ^b^	
Unknown/Not specified	26.4	27.3	30.0	29.6	6.2	
Other	5.7	0.0 ^a^	4.0 ^ab^	22.2 ^c^	6.2 ^bc^	
Consolidated Reasons for Delinquency						
Peer and social environment influence	44.7	54.5 ^a^	40.0 ^ab^	44.4 ^ab^	18.8 ^b^	<0.001 ^#^
Family-related and economicdifficulties	10.1	15.2 ^a^	8.0 ^ab^	0.0 ^b^	12.5^ab^	
Psychological reasons	6.9	3.0 ^a^	16.0 ^b^	3.7 ^ab^	0.0 ^a^	
Drug use	6.3	0.0 ^a^	2.0 ^a^	0.0 ^a^	56.2 ^b^	
Other (Unknown, not specified, possession of a weapon)	32.1	27.3 ^a^	34.0 ^ab^	51.9 ^b^	12.5 ^a^	
Family History of Incarceration						
Family members currently orpreviously incarcerated	40.3	45.5 ^a^	40.0 ^a^	11.1 ^b^	68.8 ^a^	0.001 ^#^

Values are column %. ^#^ Chi-square test. ^a,b,c^ Values having different letters were different in pairwise comparison with Bonferroni correction.

**Table 4:** Associations of Family Functioning and Impulsivity Traits With Crime Types Among Adolescents.

	Overall	Property Crimes	Crimes Against Persons	Sexual Offenses	Drug-Related Crimes	*p*
*n*	159	66	50	27	16	
Family Assessment Scale, Functional Subscale Scores (<2)						
Problem-solving < 2	43.4	45.5 ^ab^	38.0 ^ab^	63.0 ^b^	18.8 ^a^	0.031 ^#^
Communication < 2	31.4	28.8	32.0	44.4	18.8	0.316 ^#^
Roles < 2	38.4	25.8 ^a^	44.0 ^ab^	66.7 ^b^	25.0 ^a^	0.001 ^#^
Emotional responses < 2	37.1	27.3 ^a^	46.0 ^b^	51.9 ^b^	25.0 ^a^	0.047 ^#^
Showing necessary attention < 2	7.5	7.6	6.0	11.1	6.2	0.873 ^#^
Behavioral control < 2	30.2	31.8	34.0	33.3	6.2	0.179 ^#^
General functioning < 2	47.2	43.9 ^a^	44.0 ^a^	70.4 ^b^	31.2 ^a^	0.047 ^#^
Family Assessment Scale Scores						
Problem-solving	2.00 [0.83] (2.08)	2.00 [1.00] (2.07)	2.17 [0.71] (2.14)	1.83 [0.83] (1.86)	2.33 [0.75] (2.35)	0.068 *
Communication	2.11 [0.78] (2.14)	2.11 [0.78] (2.19)	2.22 [0.69] (2.1)	2.11 [1] (2.03)	2.28 [0.64] (2.26)	0.496 *
Roles	2.09 [0.64] (2.11)	2.18 [0.57] ^a^ (2.19)	2.00 [0.75] ^ab^ (2.05)	1.82 [0.64] ^b^ (1.9)	2.36 [0.70] ^a^ (2.28)	0.011 *
Emotional responses	2.17 [1.00] (2.14)	2.33 [0.92] (2.27)	2.00 [1.00] (2.08)	1.83 [1.33] (1.94)	2.08 [0.75] (2.16)	0.150 *
Showing necessary attention	2.57 [0.43] (2.554)	2.71 [0.71] ^a^ (2.66)	2.43 [0.57] ^b^ (2.49)	2.43 [0.57] ^b^ (2.41)	2.64 [0.54] ^ab^ (2.54)	0.033 *
Behavioral control	2.22 [0.67] (2.16)	2.11 [0.67] (2.15)	2.28 [0.69] (2.19)	2.11 [0.67] (2.05)	2.39 [0.33] (2.31)	0.157 *
General functioning	2.00 [0.92] (1.97)	2.08 [0.85] ^a^ (2.02)	2.08 [1.00] ^a^ (2.02)	1.50 [0.83] ^b^ (1.60)	2.17 [0.83] ^a^ (2.24)	0.002 *
Impulsive Behavior Scale Subscales						
Lack of Premeditation	22 [9.0]	21 [9.5]	22 [9.3]	21 [11.0]	24 [7.0]	0.115 *
T1	32.7	39.4	26.0	40.7	12.5	0.289 ^#^
T2	36.5	36.4	40.0	25.9	43.8	
T3	30.8	24.2	34.0	33.3	43.8	
Urgency	29 [12.0]	29 [11.3]	28.5 [14.0]	29 [12.0]	29 [9.8]	0.640 *
T1	31.4	27.3	40.0	25.9	31.2	0.765 ^#^
T2	35.2	36.4	34.0	33.3	37.5	
T3	33.3	36.4	26.0	40.7	31.2	
Sensation Seeking	28 [8.0]	28 [8.0]	25 [9.00]	29 [7.00]	28 [6.3]	0.210 *
T1	30.2	28.8	44.0	18.5	12.5	0.111 ^#^
T2	32.1	28.8	26.0	40.7	50.0	
T3	37.7	42.4	30.0	40.7	37.5	
Lack of Perseverance	22 [9.0]	22 [7.5]	18.5 [8.0]	23 [9.0]	22 [6.5]	0.412 *
T1	30.2	24.2	42.0	25.9	25.0	0.341 ^#^
T2	35.8	36.4	32.0	33.3	50.0	
T3	34.0	39.4	26.0	40.7	25.0	

Values are given as percentages, median [interquartile range, IQR], or mean. Lower scores indicate healthier family functioning. T1,T2,T3: tertile level groups. * Independent Samples-Kruskal–Wallis test; ^#^ Chi-square test. ^a,b^ Values having different letters were different in pairwise comparisons with Bonferroni correction.

**Table 5:** Estimated marginal means (95% Wald confidence intervals) of Family Assessment Scale subscales across offense types.

	Property Crimes	Crimes Against Persons	Sexual Offenses	Drug-Related Crimes	*p* *
Problem-solving	2.06 [1.9–2.21]	2.14 [1.97–2.31]	1.93 [1.68–2.19]	2.27 [1.95–2.59]	0.392
Communication	2.18 [2.06–2.3]	2.11 [1.97–2.25]	2.09 [1.89–2.3]	2.19 [1.93–2.44]	0.834
Roles	2.17 [2.06–2.28]	2.06 [1.94–2.19]	1.99 [1.81–2.17]	2.2 [1.97–2.42]	0.334
Emotional responses	2.27 [2.11–2.42]	2.09 [1.91–2.26]	1.97 [1.7–2.23]	2.12 [1.79–2.44]	0.223
Showing necessary attention	2.65 [2.56–2.75]	2.49 [2.38–2.59]	2.42 [2.26–2.58]	2.53 [2.34–2.73]	0.0499
Behavioral control	2.15 [2.05–2.25]	2.19 [2.08–2.31]	2.03 [1.87–2.2]	2.33 [2.12–2.54]	0.183
General functioning	2.02 [1.89–2.15] ^a^	2.02 [1.87–2.17] ^a^	1.65 [1.43–1.88] ^b^	2.17 [1.89–2.45] ^a^	0.021

Values are given as estimated mean [95% Wald CI]. * General linear models, Wald Chi-Square; adjusted for age, “family members currently or previously incarcerated”, adolescents’ alcohol consumption, previously attended school. ^a,b^ Values having different letters were different in pairwise comparisons with Bonferroni correction.

## Data Availability

The data presented in this study are available on request from the corresponding author. The data are not publicly available due to ethical and privacy restrictions, as they contain sensitive information from a vulnerable population.

## Abbreviations

The following abbreviations are used in this manuscript:

UPSS	Urgency, lack of Premeditation, lack of Perseverance, and Sensation Seeking
CRF	Case Report Form
FAS	Family Assessment Scale
